# Use of Detomidine Oromucosal Gel for Alleviation of Acute Anxiety and Fear in Horses: A Pilot Study

**DOI:** 10.3389/fvets.2020.573309

**Published:** 2020-10-20

**Authors:** Francesca Dai, Julia Rausk, John Aspegren, Mirja Huhtinen, Simona Cannas, Michela Minero

**Affiliations:** ^1^Dipartimento di Medicina Veterinaria, Università degli Studi di Milano, Milan, Italy; ^2^Institute of Veterinary Medicine and Animal Sciences, Estonian University of Life Sciences, Tartu, Estonia; ^3^Orion Corporation, Orion Pharma, R&D, Espoo, Finland

**Keywords:** horse, behavior, anxiety, fear, detomidine, alpha-2 agonist

## Abstract

The aim of this randomized, double-blind, placebo-controlled, parallel group clinical field study was to evaluate the effect of detomidine oromucosal gel in alleviating anxiety and fear in horses. Sixteen horses with a history of acute anxiety and fear associated with firework-related noise entered the study. On New Year's Eve, eight horses were treated with 30 μg/kg detomidine gel and eight horses with placebo gel. When fireworks were present, 75% (6/8) of the detomidine-treated horses were scored by their owners as having a good or excellent treatment effect on anxiety and fear, while 50% (3/6) of horses receiving placebo were scored to have a good effect. Horses' behavior was video-recorded and assessed with a focal animal continuous method by a treatment-blind expert observer. Results showed that when fireworks were present, walking behavior decreased significantly (*p* < 0.05) after treatment with detomidine and that horses of the placebo group, overall, showed more restlessness, vocalization, and signs of colic (Wilcoxon matched-pairs test on the first PC, *p* = 0.007). This study indicates that detomidine oromucosal gel can be used to alleviate acute noise-related anxiety and fear in horses, but larger treatment groups are needed to confirm the results.

## Introduction

As defined by Boissy (1), fear is a reaction to the perception of actual danger. Unpredictable, intermittent, and high-intensity sounds of fireworks, together with light flashes, odors, and changes in barometric pressure, may elicit fear reactions in animals (2), including horses (3). While a number of publications described the incidence of the problem in pets (2, 4–6), only few studies were published on firework-related noise anxiety in horses. Gronqvist et al. (3) reported that 79% of horses in New Zealand were rated as anxious or very anxious around fireworks by their owners, and as a consequence, 26% of horses had sustained injuries during fireworks. Reaction to fear elicits behavioral and physiological modifications (7), such as active defense (attack, menace), active flight (hiding, escape), and passive avoidance (freezing) (8); heart rate and heart rate variability variations (9–14); and rises in cortisol concentration (15–17). Horses exhibit several fear-related behaviors, including sweating, trembling, and fight responses, which may be particularly dangerous, causing severe accidents for the horse and the rider/handler (9, 18–20).

Moving the animal to a paddock away from the fireworks was reported by Gronqvist et al. (3) to be the most common management strategy adopted to mitigate this problem; the paper highlights, however, that 30% of the respondents reported that this management strategy was ineffective. In dogs and cats, several therapies are described to manage and eventually even overcome noise anxiety and fear. Generally, the condition can be treated using a system of desensitization and counterconditioning (21), but this may take several weeks; moreover, as fireworks are an occasional event, it may be difficult to set up an environment for successful habituation and desensitization. As an immediate solution during the fireworks event, medications such as sedatives and anxiolytics can be used to manage the animal's behavior (2, 21–25).

Alpha-2 adrenergic agonists, such as dexmedetomidine, have been shown to be effective against anxiety in laboratory animal models (26) and in humans (27). At subsedative doses in dogs, clonidine has been shown to be effective in alleviating acute fear-based behavior problems (28), and dexmedetomidine has been shown to be effective in alleviating acute anxiety and fear associated with noise (24). Inhibiting *locus coeruleus* firing, alpha-2 adrenergic agonists attenuate noradrenaline release from neurons in the central nervous system, producing anxiolytic and sedative/hypnotic actions (26, 27, 29).

Korpivaara et al. (24) evaluated the efficacy of dexmedetomidine oromucosal gel in reducing firework-associated acute anxiety in dogs. The results showed that dexmedetomidine was safe to use with very few adverse events reported during the study and significantly reduced anxiety and fear-related behaviors (24). The aim of the present study was to evaluate the efficacy of detomidine oromucosal gel in reducing fear-related behaviors in horses exposed to firework noise, compared with a placebo.

## Materials and Methods

The study was designed as a randomized, double-blind, placebo-controlled, clinical field study with parallel groups. Randomization of the treatment groups was conducted by an independent statistician using computer software before the start of the study.

### Animals and Facilities

The study was conducted between December 2018 and January 2019 in 16 different privately owned horse stables in Finland. Sixteen horses (eight mares and seven geldings, one stallion) of different breeds (six Finnhorses, five warmblood riding horses, three Standardbred trotters, and two ponies) aged 14.3 ± 6.0 years were included. Owners were recruited using an advertisement in the social media. Horses of both sexes and over 1 year of age with a weight between 100 and 750 kg were eligible to participate in the study. They needed to have a history of suffering from acute anxiety and fear triggered by fireworks in their stable environment. They had to spend the New Year's Eve in their own stable environment, where they had previously been exposed to firework noise. Only horses assessed by a veterinarian as class I or II (healthy or with mild systemic disease) according to the American Society of Anesthesiologists (30) could enter the study.

Exclusion criteria were as follows:

- treatments with other psychoactive medications, homeopathic remedies, pheromones, nutraceuticals or special diets to control anxiety and fear;
- behavioral training due to anxiety and fear associated with noise since last New Year's Eve;
- hypersensitivity to alpha-2 agonists or previous problems when sedated with alpha-2 agonists;
- pregnancy and/or lactation;
- strong reactions to fireworks, dangerous to itself or people around it;
- difficulties during administration of oral medications.

### Treatments

Domosedan vet. 7.6 mg/ml oromucosal gel (Orion Corporation) is an oromucosal medication containing detomidine hydrochloride as the active ingredient; the product is marketed in a prefilled, single-dose syringe enabling doses from 1.0 to 3.0 ml, with 0.25 ml increments. A placebo-prefilled syringe was prepared using the same ingredients of the active gel, except detomidine hydrochloride. Horses were randomly allocated to receive placebo (placebo; *n* = 8) or detomidine oromucosal gel 30 μg/kg (detomidine; *n* = 8). The products were prepared, labeled, and packaged so that they had identical appearance; none of the study personnel or horse owners had knowledge of the study treatment assignments of the horses. Owners administered the product sublingually; they were trained prior to the beginning of the study by showing them pictures representing the administration procedure. Owners were instructed to administer the treatment either as soon as the first, even distant, fireworks could be heard on New Year's Eve; the horse showed signs of anxiety or fear; or at the latest at 5 p.m. The treatment could be repeated up to four times, if the fireworks continued and the horse showed signs of anxiety or fear again, but not sooner than 2 h after the previous dose. The 2-h interval was chosen based on the pharmacokinetic properties of detomidine oromucosal gel in horses (31). After oromucosal administration of 40 μg/kg, the peak concentration in plasma is reached at about 1 h and 50 min and the elimination half-life is about 1 h and 15 min (31).

### Owner Assessment

#### Assessments

**Figure 1:**
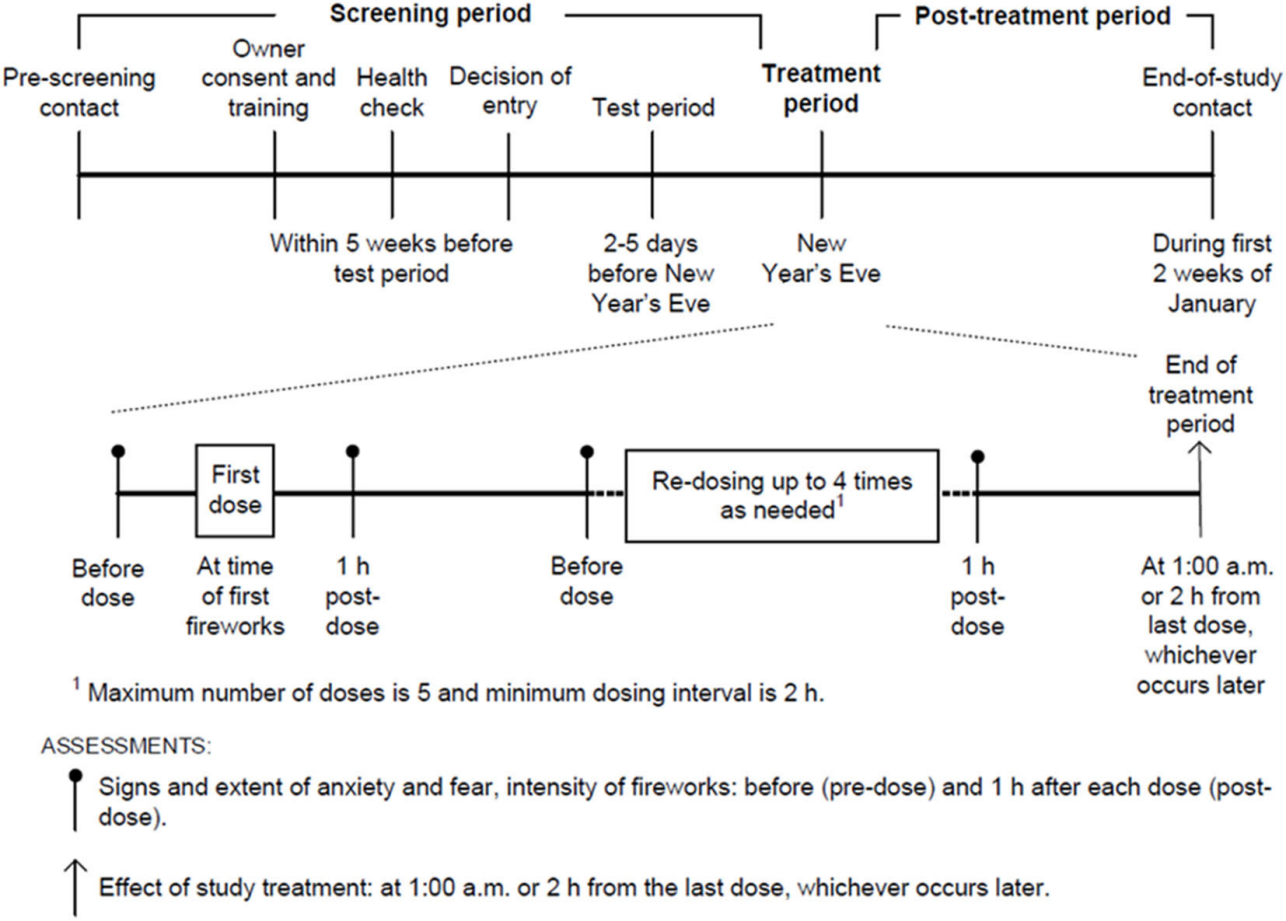
Diagram showing the study design.

The owners performed the effectiveness and safety assessments on New Year's Eve according to the study design illustrated in Figure 1.

##### Overall treatment effect (Compared with previous New Year's Eves without treatment)

The owners were treatment-blind and assessed the effect of the study treatment on horses' level of anxiety and fear, comparing their behavior to the previous New Year's Eves, when they had not been treated. The assessment was made at 1:00 a.m. or 2 h after the last dose, whichever occurred later, using the scoring scale reported in Table 1.

**Table 1:** Rating scale for the owner's assessment of the study treatment effect in their horse (overall treatment effect).

**Score**	**Description**
1	Excellent effect: The horse does not react to fireworks with anxious/fearful behavior at all.
2	Good effect: The horse's reactions are mild, and it can calm down.
3	Some effect: The horse is reacting somewhat less/milder, but it cannot calm down.
4	No effect: There is no reduction/change in the horse's reactions.
5	Worse: The horse's reaction to fireworks is stronger than before.

##### Signs and extent of anxiety and fear

The owner recorded the signs of anxiety and fear, and the extent of each sign, on New Year's Eve before dosing and 1 h after each dose. The owner observed their horse for 15 min and each of the following behavioral signs (3) was scored on a five-point scale (0 = none, 1 = only a few times, 2 = half of the time, 3 = most of the time, 4 = continuously):

- restlessness
- startle
- attempting to escape from the box
- bucking/rearing
- weaving or other stereotypic behavior
- decreased appetite
- diarrhea/increased defecation frequency
- signs of colic
- sweating
- trembling
- vocalizing
- other signs of anxiety and fear.

Owners also estimated the intensity of the fireworks during the same assessment period, on a five-point scale (1 = no fireworks, 2 = mild fireworks [distant sounds/lights], 3 = moderate fireworks [occasional sounds/lights nearby], 4 = intense fireworks [continuous/loud]).

#### Adverse Events

Adverse events, defined as any undesirable events, expected or not, occurring to the horse during the study, regardless of the event having a causal relationship to a study treatment were recorded throughout the study.

### Expert Assessment

#### Video Analysis

Videos of horses' behavior were recorded with a surveillance camera (Arlo Go with mobile internet connection) placed in their own boxes by the owner for the entire duration of the study. Video quality and camera positioning was verified remotely by the experimenter during the test period 2–5 days before the New Year's Eve. The same time points scored by the owners (pre- and post-dose) were chosen for the expert analysis. One treatment-blind animal scientist, experienced in equine behavior, performed behavioral evaluation from videos adopting a focal animal continuous recording method and using the software Solomon Coder beta 17.03.22. The ethogram used is reported in Table 2. The duration of different behaviors was recorded.

**Table 2:** Ethogram for the evaluation of horse behavior during fireworks [modified from (3)].

**Category**	**Behavior**
Restlessness^*^	Walk
	Trot on spot
	Tail switching
	Pawing
	Head shaking
Startle	Startle (horse suddenly increases the speed for a short time to get in another point of the box faster)
Attempting to escape from the box	Attempting to escape from the box
Bucking/rearing	Rear
Eat	Eat
Defecate	Defecate
Weaving or other stereotypic behavior^*^	Weaving
	Crib biting
	Licking
	Head nodding
	Pacing
	Other stereotypies
Signs of colic^*^	Roll
	Looking at the flank
	Kicking at the flank
Sweating	Sweating
Trembling	Trembling
Vocalizing^*^	Whinny
	Snorting
	Other vocalizations
Other signs of anxiety and fear^*^	Ears backwards
	Standing facing a box corner
Calm behaviors^*^	Ears forward
	Ears sideward
	Resting in a standing position (horse standing on three or four legs, head below the height of the withers, closed or half closed eyes)
	Lateral recumbency
	Sniffing
	Standing
	Looking outside
	Other

* *Behavioral categories considered for the PCA analysis*.

### Statistical Analysis

#### Owners' Assessment

Owner assessment data were collected using an electronic diary and statistical analyses were performed with SAS® for Windows version 9.4 (SAS Institute Inc., Cary, NC, USA).

The primary efficacy variable, the owner assessment of the effect of study treatment, was analyzed with a generalized linear model, cumulative logit as a link function with treatment as an explanatory variable. The maximum firework intensity score reported for each horse was used as a covariate.

As there were very mild fireworks present during the first dosing, the average of the second and third post-dosing individual signs and the extent of anxiety and fear were separately analyzed with a general linear model. Treatment was a fixed effect and baseline, i.e., average of the second and third pre-dosing individual signs, and average change from pre- to post-dosing fireworks intensity score were used as covariates.

Adverse events observed by the owner were reported descriptively by the treatment group.

#### Expert Assessment

Data from the video analyses were entered into Microsoft Excel (Microsoft Corporation, 2010, Washington, DC, USA), before being analyzed with SPSS statistical package (SPSS Statistic 25, IBM, Armonk, NY, USA). A descriptive analysis was first performed to determine mean duration and standard deviation of the different behaviors. Data were tested for normality and a matched-pairs Wilcoxon's test was used to compare the horses' behavior from pre- to the average of the second and third post-dosing in the two groups. Differences were considered to be statistically significant with *p* ≤ 0.05. Different behaviors were aggregated into larger categories, as reported in Table 2, and the average duration of the second and third post-dosing behaviors were analyzed with a multivariate statistical method, principal component analysis (PCA), to determine the role of variables and to detect common features. Factor scores were calculated for horses when the eigenvalue of the component was >1, in order to evaluate the distribution of the subjects according to the considered variables. To investigate whether the overall behavior of the horses in the two groups differed after treatment, the horses' scores on the two principal components were compared using a Wilcoxon matched-pairs test.

#### Owner and Expert Assessment Correlation on Signs and Extent of Anxiety and Fear

Percentage of time spent performing each behavior was calculated. Spearman correlation analysis has been performed to evaluate correlation between owner's and expert's behavior assessment, using SAS® for Windows version 9.4 (SAS Institute Inc., Cary, NC, USA).

### Ethical Statement

The study was conducted according to the principles of good clinical practice as defined by the International Cooperation on Harmonization of Technical Requirements for Registration of Veterinary Medicinal Products topic GL9. Informed written owner consent for the use of each animal was obtained prior to any study-specific procedures. The welfare, treatment, and care of study animals were ensured by veterinary supervision.

## Results

### Overall Treatment Effect (Owner's Assessment of the Effect of Study Treatment)

Due to weather conditions, there were no fireworks during the New Year's Eve in the environment of two horses that received placebo, which were then eliminated from the efficacy analysis. Horses in the detomidine group received one to three doses during the study, whereas horses in the placebo group received one to four doses.

In the presence of at least mild fireworks, owners assessed the treatment effect to be good or excellent (i.e., success) in 75% (6/8) of the horses in the detomidine group (30 μg/kg), whereas in the placebo group, the treatment effect was assessed to be good for 50% (3/6) and excellent for none (0%) of the horses (Table 3).

**Table 3:** Owner's assessment of the study treatment effect in their horse according to the maximum fireworks intensity.

		**Placebo (*N* = 8)**	**Detomidine 30 μg/kg (*N* = 8)**
		***n* (%)**	***n* (%)**
All horses	Excellent effect	0 (0.0)	1 (12.5)
	Good effect	4 (50.0)	5 (62.5)
	Some effect	1 (12.5)	2 (25.0)
	No effect	3 (37.5)	0 (0.0)
At least mild fireworks present	Excellent effect	0 (0.0)	1 (12.5)
	Good effect	3 (50.0)	5 (62.5)
	Some effect	1 (16.7)	2 (25.0)
	No effect	2 (33.3)	0 (0.0)
At least moderate fireworks present	Excellent effect	0 (0.0)	1 (12.5)
	Good effect	2 (40.0)	5 (62.5)
	Some effect	1 (20.0)	2 (25.0)
	No effect	2 (40.0)	0 (0.0)

In the presence of at least moderate fireworks, the proportion of horses treated with detomidine in the success category remained the same as in those exposed to at least mild fireworks, while the proportion of placebo-treated horses in the success category decreased to 40% (2/5) (Table 3).

### Signs and Extent of Anxiety and Fear (Owner Assessment)

**Figure 2:**
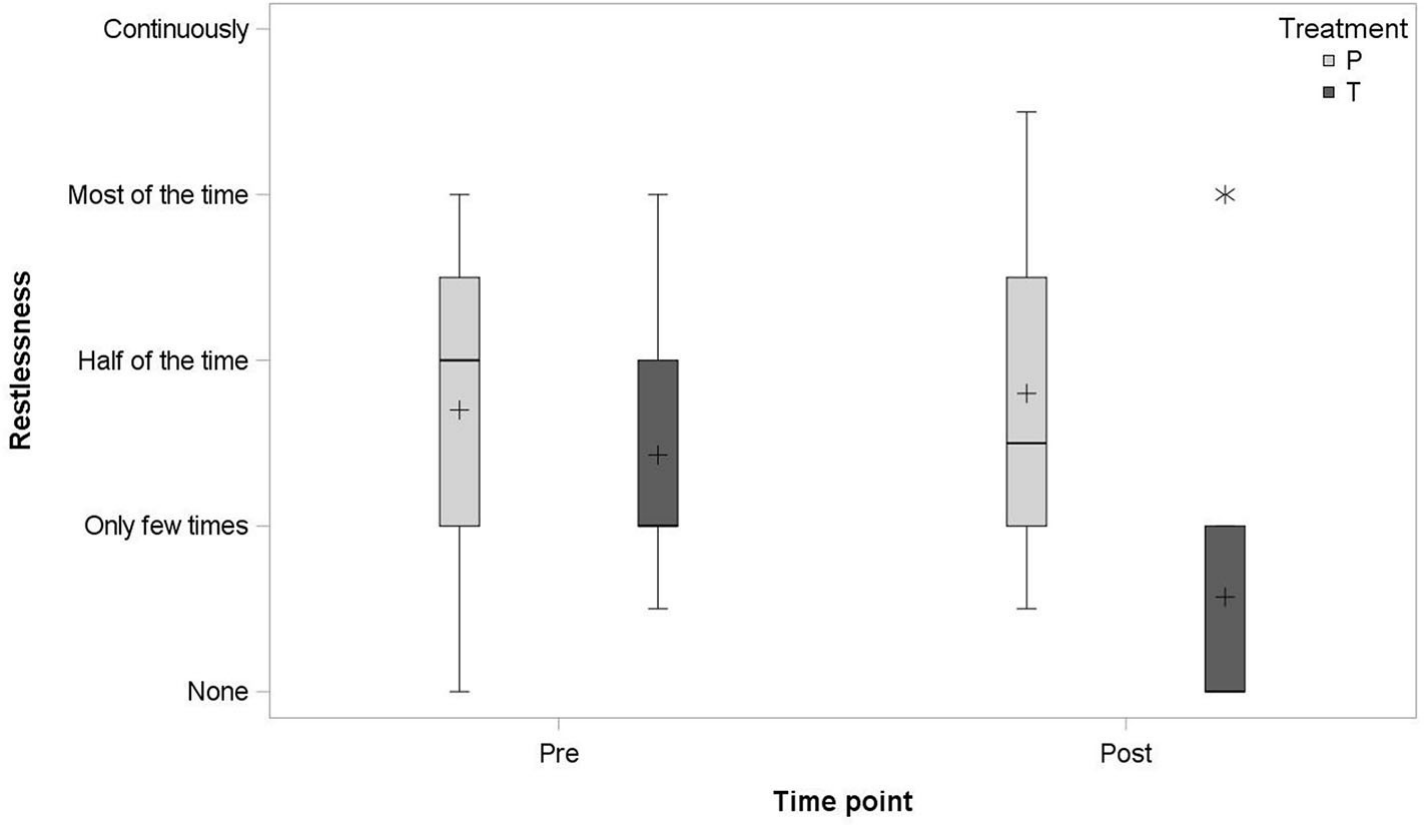
Boxplot of the average of the second and third pre- and post-dosing restlessness signs, assessed by the owners, in the detomidine group (T; *N* = 7) and the placebo group (P; *N* = 5); median (–), mean (+), and outlier (*).

No statistically significant differences between the two groups were found in any of the signs assessed by the owners. Horses treated with detomidine tended to show less signs of restlessness (*p* = 0.0548) in the presence of firework noise (Figure 2).

Weaving and other stereotypic behavior, startle, attempting to escape from the box, and diarrhea/defecation frequency were numerically lower in detomidine-treated horses than in placebo-treated horses.

### Expert Evaluation

**Figure 3:**
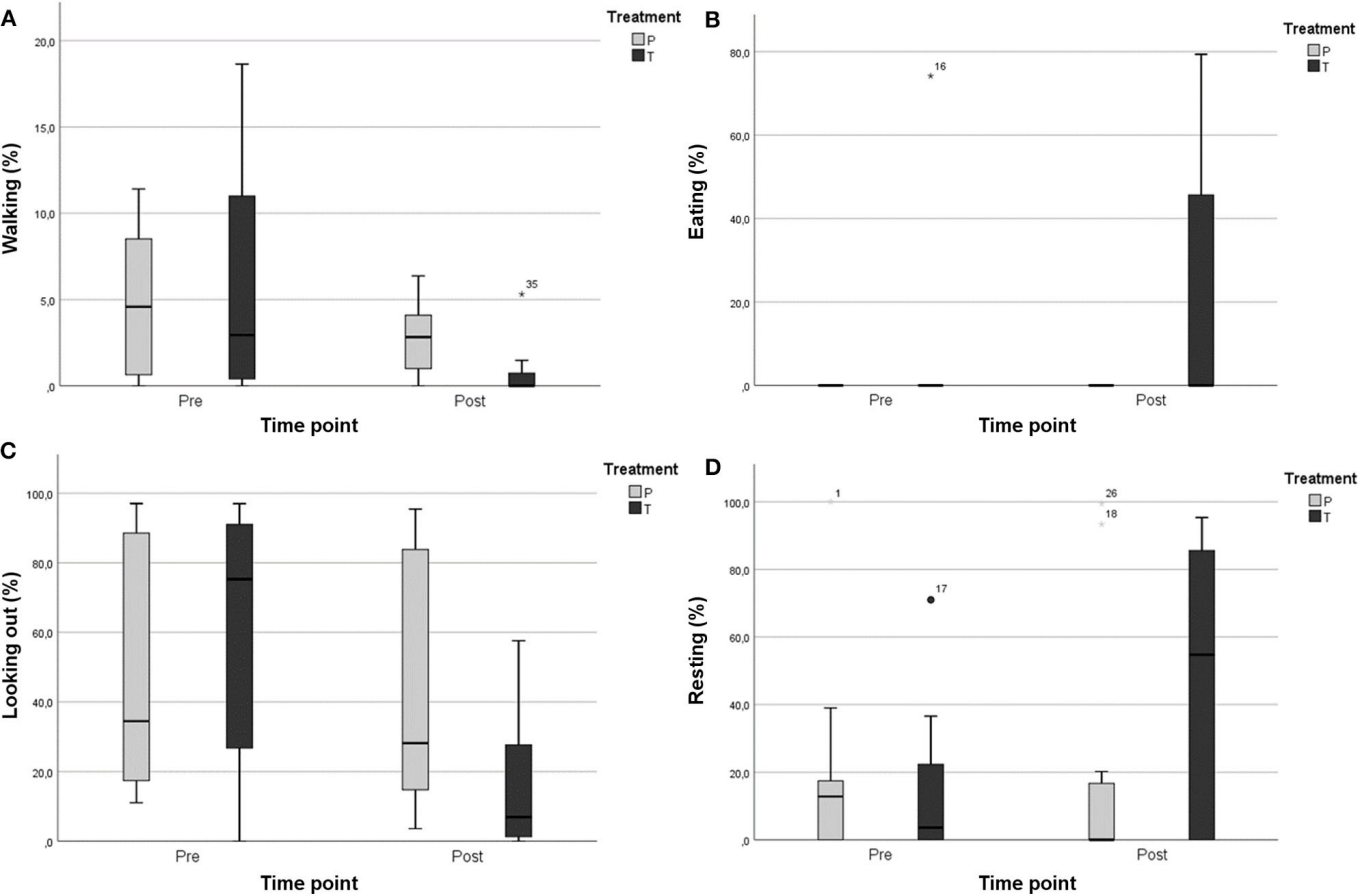
Percentage of time spent performing different behaviors in the detomidine group (T; *N* = 7) and the placebo group (*P*; *N* = 5): **(A)** walking, **(B)** eating, **(C)** looking out, and **(D)** resting. Mild outliers (°), extreme outliers (*).

All the horses in this study showed relatively few stress-related behaviors before treatment, but video analyses indicated some changes in these behaviors (Figure 3). Walking behavior decreased significantly in the detomidine group after the administration of detomidine (from 5.92 ± 7.27 of total time to 0.85 ± 1.88%), while no changes occurred in the placebo group (*p* ≤ 0.05) (Figure 3). After the administration of detomidine, horses showed more resting behavior (21.34 ± 33.27 vs. 9.27 ± 26.22%; *p* = 0.465) and eating (46.99 ± 42.53 vs. 15.36 ± 25.66%; *p* = 0.374) and spent less time looking out of the window (16.17 ± 21.24 vs. 60.37 ± 37.42%; *p* = 0.196), even though these changes were not statistically significant. Only little variation in the same behaviors occurred in horses in the placebo group (Figure 3).

The PCA revealed two underlying components, the eigenvalues of which were >1 (Table 4). These together explained 61.27% of the variation.

**Table 4:** Principal component analysis (PCA) of quantitative data calculated from correlation matrix.

**Component**	**Eigen value**	**Explained variance %**	**Cumulative explained variance %**
1	3.098	38.724	38.724
2	1.804	22.550	61.274
**Behaviors**		**Component**	
		**PC1 loadings**	**PC2 loadings**
Restlessness		0.830	0.387
Calm behavior		−0.671	0.443
Other anxiety		0.127	−0.735
Vocalizing		0.809	0.394
Signs of colic		0.696	−0.103
Stereotypic		0.511	0.510
Eat		−0.085	−0.772
Defecate		0.199	0.624

**Figure 4:**
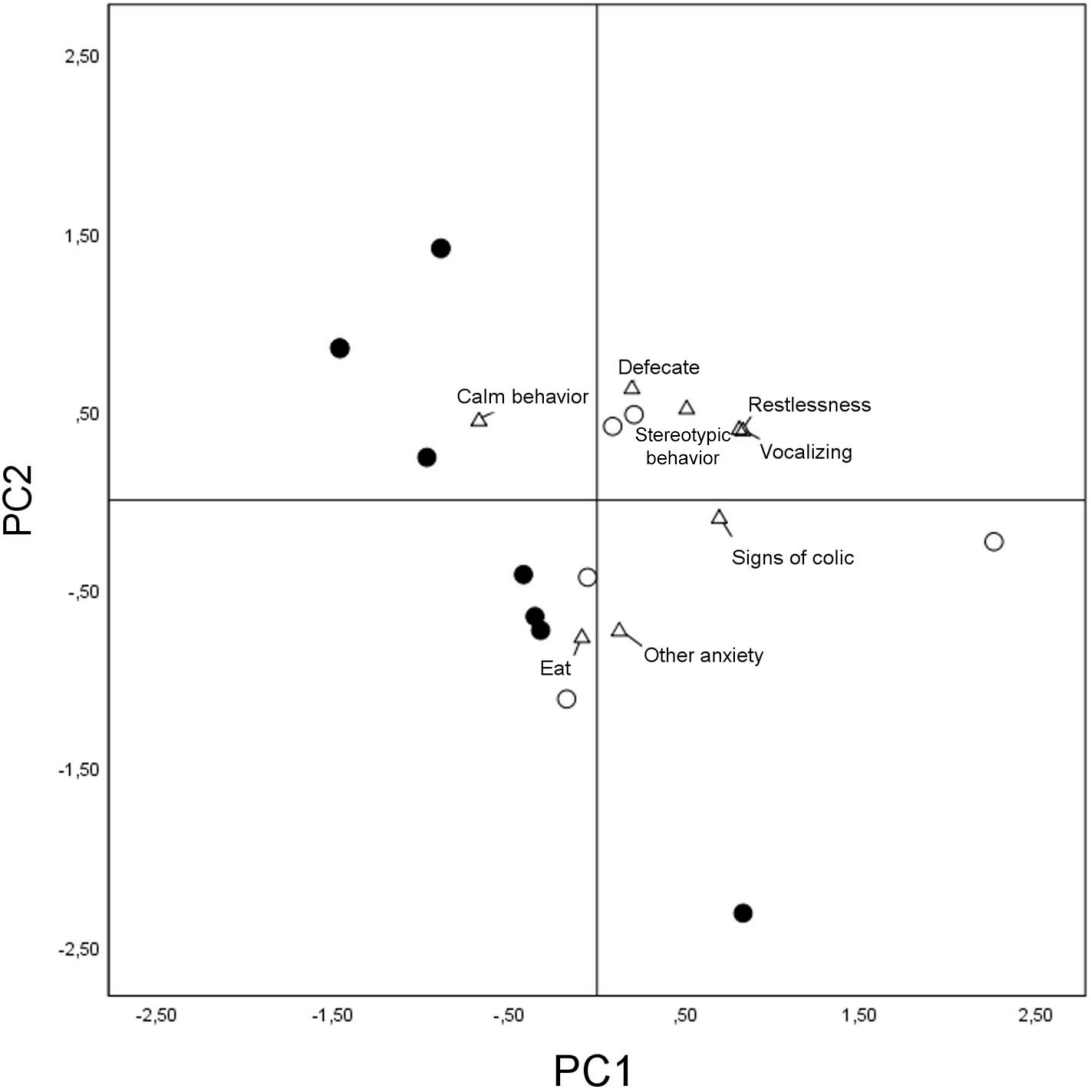
Loadings plot of the behavioral variables considered for the first and second principal components and scores plot for the two groups of horses. Detomidine-treated horses (*N* = 7) are marked with dots and placebo-treated horses (*N* = 5) with circles in terms of the first and second principal components. Behaviors are marked with triangles.

The first component (PC1) seemed to be associated with the stress level, showing positive loadings for restlessness, vocalizing, signs of colic, and stereotypic behavior and negative loadings for calm behavior. The second component (PC2), showing positive loadings for the variable “defecate” and negative loadings for “other anxiety” and “eat” (Table 4), seemed more difficult to interpret. Horses' PC scores are plotted in Figure 4 showing that they did not gather homogeneously, with subjects of the group treated with detomidine clustering in the quadrant of the graph referring to eating and calm behaviors.

The Wilcoxon matched-pairs test provided evidence of a significant difference in the behavior of the horses on the first principal component, indicating that placebo-treated horses, overall, showed more restlessness, vocalization, and signs of colic (*p* = 0.007).

### Owner and Expert Assessment Correlation on Signs and Extent of Anxiety and Fear

Statistically significant Spearman correlation (*r*_s_) between owner's and expert's assessment was seen for some of the signs: weaving or other stereotypic behavior (*r*_s_ = 0.39, *p* = 0.0216) and for restlessness (*r*_s_ = 0.48, *p* = 0.0036).

### Adverse Events

There were no serious adverse events or premature study treatment discontinuations due to adverse events. No accidental human exposure was reported. Only one adverse event was reported after the start of study treatment. One of the horses started sweating mildly 20 min after the first 30 μg/kg detomidine administration.

## Discussion

The objective of this pilot study was to evaluate the efficacy and clinical safety of detomidine against placebo for alleviation of acute anxiety and fear associated with firework-related noise in horses. Only a single mild adverse effect (sweating) was reported in one horse after administration of the first dose and this could suggest that detomidine is safe to use for alleviating horses' fear during fireworks.

Our results indicate a beneficial effect of detomidine on horses' signs of acute anxiety and fear associated with fireworks; although no statistically significant differences were found, owners tended to assess the treatment effect to be excellent or good more frequently for horses treated with detomidine than with placebo. The fact that horses were assessed by the expert as eating more after detomidine administration and not being apathetic indicates an anxiolytic rather than a sedative effect. Expert evaluation revealed that relatively few stress-related behaviors had been shown before treatment, probably because horses with strong reactions to fireworks were not included in the study for ethical reasons. After being treated with detomidine, horses appeared in general less stressed, resting and eating more, and spending less time looking out of the window, even though the only behavior that showed a significant decrease was walking in the box. These results confirm that horses benefit clinically from this treatment. It can, however, be expected that the significance level of these initial findings would be increased when employing a higher sample size. It is worth noticing that 50% of the horses in the placebo group were considered by their owners as being less frightened after placebo administration. Placebo effect has also been reported in dogs treated with dexmedetomidine, even though the incidence was lower (24). In clinical studies, the expectation of a response can influence subjective interpretation. However, behavioral complaints are often dependent on owner perception (i.e., defined subjectively). The expectation of a response, giving treatment to an animal, as well as assessment-related and documentation-related activities probably influenced the assessment of the horse owners. However, our results suggest that owners are capable of identifying restlessness and stereotypic behavior of their horses. Even if it is important to keep in mind that it is the owner's impression of improvement that finally determines if a treatment will be applied (32), an expert behavioral evaluation should be acquired when assessing the efficacy of a pharmacological treatment for behavioral disorders.

Limitations of this study are the relatively small sample size, possible uneven fireworks intensity, and a horse selection process based mainly on owners' reports. Despite these limitations, this pilot study represents a starting point providing relevant preliminary information regarding the use of detomidine in horses undergoing situations eliciting acute fear and anxiety. Further research is needed to confirm these preliminary results and should not only include a larger sample size but also consider physiological indicators, such as heart rate, heart rate variability, or cortisol concentration, contributing to a more in-depth evaluation of fear and anxiety intensity. In conclusion, our results suggest that detomidine oromucosal gel could be effective in alleviating acute fear and anxiety triggered by firework-related noise in horses, without inducing adverse effects.

Since many horse owners perceive firework-related noise anxiety to be a health concern for their animals, it is crucial that owners should consult their veterinarian to identify the best management strategy for their horses. The results of this pilot study suggest that anxiety-associated sympathetic arousal caused by noradrenaline, manifesting in fear and anxiety behaviors, was successfully counteracted by the study treatment in most cases. It is also noteworthy that horses already showing signs of fear and anxiety benefitted from detomidine gel treatment. This was evident as redosing of the study medication was only permitted when the horse began to show signs of fear and anxiety again. The use of detomidine oromucosal gel at a lower than the label dose and giving it repeatedly as needed during the New Year's Eve offers a feasible solution for horses when management practices are not enough to counteract fear and anxiety caused by fireworks.

## Data Availability Statement

The raw data supporting the conclusions of this article will be made available by the authors, without undue reservation.

## Ethics Statement

The study was conducted in Finland and the clinical trial application was approved by the Finnish Medicine Agency (Fimea). The Directive 2010/63/EU on the protection of animals used for scientific purpose shall not apply to veterinary clinical trials required for the marketing authorization of a veterinary medicinal product. Therefore, no Ethical Committee approval was needed. The study was conducted according to the principles of good clinical practice as defined by the International Cooperation on Harmonization of Technical Requirements for Registration of Veterinary Medicinal Products topic GL9. Informed written owner consent for the use of each animal was obtained prior to any study-specific procedures. The welfare, treatment and care of study animals were ensured by veterinary supervision. Written informed consent was obtained from the owners for the participation of their animals in this study.

## Author Contributions

FD: methodology, data curation, and writing—original draft. JR: investigation and writing—original draft. JA: formal analysis, data curation, and writing. MH: conceptualization, methodology, project administration, and writing. SC: formal analysis and writing—review and editing. MM: conceptualization, methodology, supervision, and writing—review and editing. All authors contributed to the article and approved the submitted version.

## Conflict of Interest

MH and JA are employees of Orion Corporation. The remaining authors declare that the research was conducted in the absence of any commercial or financial relationships that could be construed as a potential conflict of interest. The authors declare that this study received funding from Orion Corporation. The funder had the following involvement with the study: JA: formal analysis, data curation, and writing. MH: conceptualization, methodology, project administration, and writing.

## References

[1] Boissy A Fear fearfulness in determining behavior. In: Grandin T. editor. Genetics and the Behaviour of Domestic Animals. San Diego: Academic Press (1998). p. 67–111.

[2] Mills DS. A double-blind placebo-controlled study into the efficacy of a homeopathic remedy for fear of firework noises in the dog (Canis familiaris). Vet. J. (2008) 177:80–88. 10.1016/j.tvjl.2007.04.007 17572119

[3] Gronqvist G Rogers C Gee E. The management of horses during fireworks in New Zealand. Animals. (2016) 6:20. 10.3390/ani6030020 27005667 PMC4810048

[4] Blackwell EJ Bradshaw JWS Casey RA Fear responses to noises in domestic dogs: Prevalence, risk factors and co-occurrence with other fear related behaviour. Appl Anim Behav Sci. (2013) 145:15–25. 10.1016/j.applanim.2012.12.004

[5] Dale AR Walker JK Farnworth MJ Morrissey SV Waran NK. A survey of owners' perceptions of fear of fireworks in a sample of dogs and cats in New Zealand. N Z Vet J. (2010) 58:286–91. 10.1080/00480169.2010.69403 21151214

[6] Overall KL Dunham AE Juarbe-Diaz SV Phenotypic determination of noise reactivity in 3 breeds of working dogs: a cautionary tale of age, breed, behavioral assessment, and genetics. J Vet Behav Clin Appl Res. (2016) 16:113–25. 10.1016/j.jveb.2016.09.007

[7] Forkman B Boissy A Meunier-Salaün MC Canali E Jones RB. A critical review of fear tests used on cattle, pigs, sheep, poultry and horses. Physiol Behav. (2007) 92:340–74. 10.1016/j.physbeh.2007.03.016 18046784

[8] Erhard H Mendl M Tonic immobility and emergence time in pigs - more evidence for behavioural strategies. Appl Anim Behav Sci. (1999) 61:227–37. 10.1016/S0168-1591(98)00196-8

[9] Christensen JW Malmkvist J Nielsen BL Keeling LJ. Effects of a calm companion on fear reactions in naïve test horses. Equine Vet J. (2008) 40:46–50. 10.2746/042516408X245171 18083659

[10] Momozawa Y Ono T Sato F Kikusui T Takeuchi Y Mori Y Assessment of equine temperament by a questionnaire survey to caretakers and evaluation of its reliability by simultaneous behavior test. Appl Anim Behav Sci. (2003) 84:127–38. 10.1016/j.applanim.2003.08.001

[11] Rietmann T Stuart A Bernasconi P Stauffacher M Auer J Weishaupt M Assessment of mental stress in warmblood horses: heart rate variability in comparison to heart rate and selected behavioural parameters. Appl Anim Behav Sci. (2004) 88:121–36. 10.1016/j.applanim.2004.02.016

[12] Stewart M Stafford KJ Dowling SK Schaefer AL Webster JR Eye temperature and heart rate variability of calves disbudded with or without local anaesthetic. Physiol Behav. (2008) 93:789–97. 10.1016/j.physbeh.2007.11.044 18177678

[13] Visser EK van Reenen CG van der Werf JTN Schilder MBH Knaap JH Barneveld A. Heart rate and heart rate variability during a novel object test and a handling test in young horses. Physiol Behav. (2002) 76:289–96. 10.1016/S0031-9384(02)00698-4 12044602

[14] von Borell E Langbein J Després G Hansen S Leterrier C Marchant-Forde J. Heart rate variability as a measure of autonomic regulation of cardiac activity for assessing stress and welfare in farm animals - A review. Physiol Behav. (2007) 92:293–316. 10.1016/j.physbeh.2007.01.007 17320122

[15] Anderson M Friend T Evans J Burshong D Behavioral assessment of horses in therapeutic riding programs. Appl Anim Behav Sci. (1999) 63:11–24. 10.1016/S0168-1591(98)00237-8

[16] Cook N Schaefer A Warren L Burwash L Anderson M Baron V Adrenocortical and metabolic responses to ACTH injection in horses: an assessment by salivary cortisol and infrared thermography of the eye. Can J Anim Sci. (2001) 81:621.

[17] Flauger B Krueger K Gerhards H Möstl E. Simplified method to measure glucocorticoid metabolites in faeces of horses. Vet Res Commun. (2010) 34:185–95. 10.1007/s11259-010-9344-y 20182914

[18] Christensen JW Keeling LJ Nielsen BL Responses of horses to novel visual, olfactory and auditory stimuli. Appl Anim Behav Sci. (2005) 93:53–65. 10.1016/j.applanim.2005.06.017

[19] McGreevy P McLean A Equitation Science. West Sussex: Wiley-Blackwell, Chichester (2010).

[20] McGreevy PD Henshall C Starling MJ McLean AN Boakes RA The importance of safety signals in animal handling and training. J Vet Behav Clin. Appl Res. (2013) 9:382–7. 10.1016/j.jveb.2014.06.006

[21] Overall K. Noise phobias in dogs. In: Horwitz D, Mills D, Heath S. editors. BSAVA Manual of Canine and Feline Behavioural Medicine. Gloucester, UK: BSAVA (2002). p. 164–72.

[22] Mills DS Estelles MG Coleshaw PH Shorthouse C Park R Ln L Retrospective analysis of the treatment of firework fears in dogs Accidental electroshock of fish in a recirculation facility. Vet Rec. (2003) 153:561–2. 10.1136/vr.153.18.561 14627237

[23] Mills D Management of noise fears and phobias in pets. In Pract. (2005) 27:248–55. 10.1136/inpract.27.5.248

[24] Korpivaara M Laapas K Huhtinen M Schöning B Overall K. Dexmedetomidine oromucosal gel for noise-associated acute anxiety and fear in dogs-a randomised, double-blind, placebo-controlled clinical study. Vet Rec. (2017) 180:356. 10.1136/vr.104045 28213531

[25] Engel O Müller HW Klee R Francke B Mills DS. Effectiveness of imepitoin for the control of anxiety and fear associated with noise phobia in dogs. J Vet Intern Med. (2019) 33:2675–84. 10.1111/jvim.15608 31568622 PMC6872611

[26] Millan MJ Dekeyne A Newman-Tancredi A Cussac D Audinot V Milligan G S18616, a highly potent, spiroimidazoline agonist at α2-adrenoceptors: I. Receptor profile, antinociceptive and hypothermic actions in comparison with dexmedetomidine and clonidine. J Pharmacol Exp Ther. (2000) 295:1192–205. Available online at: https://jpet-aspetjournals-org.eu1.proxy.openathens.net/content/jpet/295/3/1192.full.pdf 11082457

[27] Mantz J Alpha2-adrenoceptor agonists: Analgesia, sedation, anxiolysis, haemodynamics, respiratory function and weaning. Bailliere's Best Pract Res Clin Anaesthesiol. (2000) 14:433–48. 10.1053/bean.2000.0094

[28] Ogata N Dodman NH The use of clonidine in the treatment of fear-based behavior problems in dogs: an open trial. J Vet Behav Clin Appl Res. (2011) 6:130–7. 10.1016/j.jveb.2010.10.004

[29] Tanaka M Yoshida M Emoto H Ishii H. Noradrenaline systems in the hypothalamus, amygdala and locus coeruleus are involved in the provocation of anxiety: Basic studies. Eur J Pharmacol. (2000) 405:397–406. 10.1016/S0014-2999(00)00569-0 11033344

[30] Pasternak L Arens J Caplan R Connis R Fleisher L Flowerdew R Practice advisory for preanesthesia evaluation: A report by the American Society of Anesthesiologists Task Force on preanesthesia evaluation. Anesthesiology. (2002) 96:485–96. 10.1097/00000542-200202000-00037 11818784

[31] Kaukinen H Aspegrén J Hyyppa S Tamm L Salonen J. Bioavailability of detomidine administered sublingually to horses as an oromucosal gel. J Vet Pharmacol Ther. (2010) 34:76–81. 10.1111/j.1365-2885.2010.01193.x 21219348

[32] Levine ED Ramos D Mills DS A prospective study of two self-help CD based desensitization and counter-conditioning programmes with the use of Dog Appeasing Pheromone for the treatment of firework fears in dogs (Canis familiaris). Appl Anim Behav Sci. (2007) 105:311–29. 10.1016/j.applanim.2006.11.006

